# Integrated study of miR‐215 promoting breast cancer cell apoptosis by targeting RAD54B

**DOI:** 10.1111/jcmm.16402

**Published:** 2021-02-26

**Authors:** Mingyuan Wang, Jingnan Liao, Chang Tan, Hong Zhou, Jinjin Wang, Kangkai Wang, Yiming Li, Wei Wu

**Affiliations:** ^1^ Department of Pathophysiology School of Basic Medical Science Central South University Changsha China; ^2^ Department of Gynaecology the Affiliated Zhuzhou Hospital Xiangya Medical College Central South University Zhuzhou China; ^3^ Institute of Reproductive and Stem Cell Engineering School of Basic Medical Science Central South University Changsha China; ^4^ Key Laboratory of Sepsis Translational Medicine of Hunan Central South University Changsha China; ^5^ Department of Laboratory Animals Hunan Key Laboratory of Animal Models for Human Diseases Xiangya School of Medicine Central South University Changsha China; ^6^ Department of Geratic Surgery Xiangya Hospital Central South University Changsha China; ^7^ National Clinical Research Center for Geriatric Disorders Xiangya Hospital Central South University Changsha China

**Keywords:** biomarker, breast cancer, integrated analysis, miR‐215‐5p, RAD54B

## Abstract

**Background:**

MicroRNAs (miRNAs) are widely distributed in cells and participate in the regulation of the pathophysiological process of many diseases. As an important part of non‐coding RNA, miRNAs regulate a variety of molecules and signal pathways in tumour cells. However, the evidence for regulatory mechanisms of specific miRNAs in tumour cells is still lacking.

**Methods:**

In this study, we used transcriptomics analysis and integrated a variety of public databases to screen miRNAs that have key regulatory effects on breast cancer (BC). In addition, we used in vitro and in vivo studies and combined clinical samples to verify its regulatory mechanism.

**Results:**

We found that among the specific miRNAs, miR‐215‐5p is a key regulator in BC. Compared with normal adjacent tissues, miR‐215‐5p has a lower expression level in BC tissues. Patients with high expression levels of miR‐215‐5p have a longer survival time. miR‐215‐5p can specifically target the 3′UTR region of RAD54B mRNA and down‐regulate the expression of RAD54B, thereby inhibiting the proliferation of BC cells and promoting the apoptosis of BC cells.

**Conclusions:**

Finally, we found that miR‐215‐5p can be used as an important biomarker for BC. We have clarified its function and revealed its mechanism of targeting RAD54B mRNA for the first time. This may provide important clues to reveal the deeper molecular regulation mechanism of BC.

## 1 INTRODUCTION

Breast cancer (BC) is the most common female tumour in the world.^1^ Its high morbidity and high mortality have attracted the attention of clinicians and scientific researchers. It is a heterogeneous disease, and current researches have found that its occurrence and development were related to numerous genetic changes.^2^, ^3^, ^4^ A large number of biologically active molecules participate in the regulation of signalling pathways in BC cells. However, tumour cells have many phenotypes and their intracellular molecular regulation mechanisms are complex. Screening and research on key factors is still scarce. Therefore, more comprehensive research is needed to clarify the mechanism of action of key regulatory factors in tumour cells.

MicroRNA (miRNA) is a type of non‐coding single‐stranded RNA with a length of about 22 nucleotides expressed by endogenous genes, which can be complementary to the 3′UTR region of the target mRNA to inhibit protein translation and participate in post‐transcription regulation of gene expression.^5^ In recent years, miRNAs have become more and more important in tumour research as epigenetic regulatory factors.^6^, ^7^ In different blood samples or solid tumour tissues, miRNAs show a high degree of specificity and are closely related to clinical aspects, such as the survival time of tumour patients, drug sensitivity and mRNA expression. A number of studies have found that miRNAs have an inevitable connection with the occurrence and development of BC.^8^, ^9^, ^10^ This indicates that miRNAs have important regulatory functions. On the other hand, it shows that miRNAs have a wide range of functions and complex regulatory relationships. Therefore, the mechanism of the key miRNA needs to be clarified urgently.

Our previous studies have found that among the numerous genetic changes in tumour cells, there were hub‐genes that played key roles.^11^, ^12^ With the wide application of high‐throughput and multi‐omics research, the screening of key factors in tumour cells has continued to deepen, providing important clues for in vitro and in vivo experiments.

In this study, we focused on combining multiple public databases to analyse key miRNAs in BC. Through in vitro and in vivo experiments and clinical patient samples, its molecular regulation mechanism was explored in multiple aspects. We identified that miR‐215‐5p has a significant inhibitory effect on the proliferation of BC cells. MiR‐215‐5p inhibits the protein expression of RAD54B by specifically binding the 3′UTR of RAD54B mRNA, thereby inducing apoptosis of BC cells. We have clarified the correlation between miR‐215‐5p and RAD54B, revealed the molecular regulation mechanism in BC cells and provided a reference for the research of BC targeted therapy.

## 2 MATERIALS AND METHODS

### 2.1 Data procession

The gene expression microarray data of BC tissues of clinical patients and BC cell lines were obtained from the MERAV (MERAV, http://merav.wi.mit.edu) database.^13^ After log2 conversion, all the original data were used for subsequent analysis.

### 2.2 Differential gene expression analysis

The gene expression data were divided into two groups: breast cancer cell lines and normal breast cells. The R package ‘limma’ was used for analysis of differentially expressed genes. Volcano map was used to show the difference between the two groups of genes. The criteria used for screening were log_2_ (fold‐change) greater than 1 and false discovery rate (FDR) less than or equal to 0.05. Afterwards, the highly expressed genes in tumour cells were extracted for subsequent analysis.

### 2.3 Establishment of weighted co‐expression network analysis

In order to comprehensively explore the hub‐gene in BC, we collected the gene expression data and corresponding clinical information of 361 BC patients from the MERAV database. After all data filtering and normalized, the top 25% most variant genes were then selected for subsequent weighted gene co‐expression network analysis (WGCNA) analysis.

The WGCNA package in R software was used to study the correlation patterns between genes as previously described,^14^ and the gene modules related to clinical information were obtained. Pearson correlation coefficient between genes were calculated, and a weighted adjacency matrix a_mn_ = |C_mn_|^β^ was constructed, where a_mn_ represents the adjacency coefficient between gene m and gene n; C_mn_ represents the Pearson correlation coefficient between gene m and gene n. The scale‐free network was ensured by selecting the soft threshold *β* = 7, and the minimum module size was set to 30 genes. Then, we identified highly similar modules through clustering and merged the similar modules with a height cut threshold of 0.25. We used Pearson correlation analysis to calculate the correlation coefficient and *P* value between the characteristic gene of the gene module and the clinical information, and used the labelled heat map function to vividly display it in the form of heat map. We took the most relevant gene modules as the research object to find the hub‐gene with biological significance.

### 2.4 Gene function enrichment and protein‐protein interaction (PPI) network construction

In order to narrow the scope of hub‐gene screening, we selected the intersection of the differentially highly expressed genes and the gene modules with the most significant clinical phenotypes for subsequent analysis.

By using g:Profiler (https://biit.cs.ut.ee/gprofiler/gconvert.cgi),^15^ the intersection genes were enriched into five categories: molecular function (MF), cellular component (CC), biological process (BP), Kyoto Encyclopedia of Genes and Genomes (KEGG) pathway and WikiPathways (WP). Results were demonstrated in the form of network diagrams.

PPI networks were extracted from the STRING database (http://string‐db.org/)^16^ and visualized using Cytoscape software. Subsequently, we used the MCODE plug‐in to extract sub‐networks containing key nodes in the PPI network.

### 2.5 miRNA enrichment and multi‐database integration analysis

Next, we used g:Profiler to enrich miRNAs targeting candidate genes. The relevant parameters were selected by default. The networks of miRNAs with candidate target genes were displayed using Cytoscape software.

The Kaplan–Meier methodology was used to construct overall survival curves for candidate miRNAs as mentioned in previous research.^17^ The relevant construction parameters adopt the system default. The online analysis tool UALCAN (http://ualcan.path.uab.edu) was used to analyse the BC data in TCGA.^18^

### 2.6 Cell culture

Human breast cancer cell line (Michigan Cancer Foundation‐7, MCF‐7) and human normal breast epithelial cell line (MCF‐10A) were purchased from American Type Culture Collection (USA). Cells were cultured in RPMI1640 medium containing 10% foetal bovine serum (Gibco), 100 μg penicillin and 100 U/mL streptomycin. Cells were cultured at 37°C in a humidified environment with 5% CO_2_.

### 2.7 Transfection and adenovirus transduction

As described by Jiang et al in previous research.^19^ For enhanced or decreased expression of miR‐215, miRNA mimics control (mimics NC), miR‐215 mimics, miRNA inhibitor control (inhibitor NC), or miR‐215 inhibitor were purchased from GenePharma. miRNAs were transfected into cells using Lipofectamine 2000 reagent (Thermo Fisher Scientific) according to the manufacturer's instructions. After 24 hours, cells were harvested for subsequent analysis.

The adenovirus vector constructed by UCBio (Changsha, China) was used for stable overexpression or knockdown of RAD54B in cells according to the manufacturer's instructions, and selected with puromycin (Sigma‐Aldrich) for 2‐3 weeks to obtain stable cell lines.

### 2.8 Preparation of the clinical samples of BC

Twelve BC patients hospitalized in the affiliated Zhuzhou hospital Xiangya medical college from 1st January 2019 to 1st January 2020 were randomly selected. The BC and corresponding adjacent tissues were well‐preserved. The Ethics committee of the affiliated Zhuzhou hospital Xiangya medical college approved the research protocol.

### 2.9 Quantitative real‐time PCR (qRT‐PCR)

Total RNA was isolated from tissues or cells using TRIzol reagent (TaKaRa) and reverse transcribed with RT‐for‐PCR Kit (TaKaRa). And we used polyA tailing method to reverse transcription of miRNA. RT products were used as templates for quantitative real‐time PCR (qRT‐PCR) with specific primers. The primers were designed using NCBI online tool Primer‐BLAST (www.ncbi.nlm.nih.gov/tools/primer‐blast). The following primers were used in this study: RAD54B, forward 5′‐ACGACCAGATAAGAATCACCA‐3′ and reverse 5′‐TCAGCAAGAATAGCTCCACA‐3′; miR‐215‐5p, forward 5′‐ATGACCTATGAATTGACAGAC‐3′; U6, forward, 5′‐CTCGCTTCGGCAGCACA‐3′; GAPDH, forward 5′‐CCTTCCGTGTCCCCACT‐3′ and reverse 5′‐GCCTGCTTCACCACCTTC‐3′. The mRNA level of the target gene was analysed by SYBR Premix EX Taq (TaKaRa) (n = 3, each in triplicates) and ABI PRISM 7900 Real‐Time PCR System (Applied Biosystems). Glyceraldehyde 3‐phosphate dehydrogenase (GAPDH) was used as an internal control for mRNA. U6 was used as an internal control for miRNA. The expression level of the target gene was determined using 2^−ΔΔCT^ method.

### 2.10 Cell proliferation assay

The 3‐(4,5‐dimethyl‐2‐thiazolyl)‐2, 5‐diphenyl‐2H‐tetrazolium bromide (MTT) method was used to detect cell proliferation. The cells in the logarithmic growth phase were inoculated into 96‐well culture plates, and the cell density was 5 × 10^4^/mL, 100 μL/plate of suspension per well. Cells were then transfected/transduced with miRNA or adenovirus. A blank group was set up with only cell culture medium added, and each group repeated three wells. The total volume of each well was 200 μL. After 24 hours of cell culture, 5 mg/mL MTT 20 μL was added and incubated at 37°C for 4 hours. 150 μL of DMSO was added to each well to dissolve and shake for 10 minutes. After the crystals were fully dissolved, the optical density D (λ) of each hole was measured at a wavelength of 490 nm using a microplate reader (Menlo Park), and the inhibition rate of cell proliferation was calculated.

### 2.11 Wound scratch healing assay

We selected cells in the logarithmic growth phase and adjusted the cell density to 5 × 10^5^ cells per millilitre. Then, we added the cells to the 6‐well plate for 10^6^ cells per well. After 24 hours of cultured in a cell incubator, the cells formed a monolayer. We used a 200 μL sterile pipette tip to draw a straight line perpendicular to the bottom of the plate and washed off the exfoliated cells with phosphate buffered saline (PBS) solution. Pictures were taken by an inverted microscope on 1, 3 and 5 days, and calculated the cell migration rate. Cell migration rate = migration distance/original distance × 100%.

### 2.12 Cell apoptosis assay

After trypsinization, the cells were collected and centrifuged at 1000 r/min for 10 minutes. Cell apoptosis assay was performed using Annexin‐VFITC/PI Apoptosis Assay Kit (Beyotime) according to the manufacturer's instruction. Finally, we used a flow cytometer (BD FACSCalibur) to detect.

### 2.13 Western blotting and immunofluorescence (IF)

Western blotting was used to detect protein expression levels. The cells were seeded in a 6‐well plate at a density of 5 × 10^5^/well, and the cell protein was extracted with RIPA protein lysate (including PMSF) on ice. The protein sample was electrophoresed on an SDS‐PAGE gel and transferred to a PVDF membrane. The concentration of the primary antibody was 1:1000. The next day, the secondary antibody (1:5000) was added and incubated for 1 hour at room temperature. Finally, the membranes were visualized in ECL luminescent solution, and the pictures were obtained on the Bio‐Rad imager. ImageJ software was used for analysis.

The cells seeded in the 24‐well plate were washed three times with PBS, fixed with paraformaldehyde and permeabilized by Triton X‐100. After the cells were blocked by donkey serum, the primary antibody (1:100) was added and incubated overnight at 4°C. On the second day, the diluted secondary antibody (1:100) was added. Finally, it was stained with 4′,6‐diamidino‐2‐phenylindole (DAPI) for 15 minutes and washed with PBS for three times. The stained cells were visualized using an Olympus BX61 microscope (Olympus Corporation). Antibodies used were anti‐RAD54B (ab222891 and ab83311; Abcam), anti‐Bax (ab3191; Abcam), anti‐Bcl‐2 (ab194583; Abcam) and anti‐GAPDH (ab8245; Abcam).

### 2.14 Dual luciferase reporter assay

The WT and mutant 3′‐untranslated region (3′‐UTR) of RAD54B were designated as pmirGLO‐RAD54B‐WT and pmirGLO‐RAD54B‐MUT. The construct was cotransfected with mimic NC, miR‐215 mimic, inhibitor NC and miR‐215 inhibitor into MCF‐7 cells, respectively. After 48 hours of culture, the fluorescence activity of MCF‐7 cells was detected. The luciferase activity of each sample was normalized to the Renilla luciferase activity. All experiments were conducted three times.

### 2.15 Tumour xenograft experiments

The procedures for all animal experiments were approved by the Ethical Committee for Animal Research of Central South University. Male nude mice were obtained from Shanghai Experimental Animal Center. MCF‐7 cells were injected subcutaneously into the lower left flank of nude mice to establish the tumour xenograft model. One week later, miR‐215 agomir was injected into the tumour every three days at a dose of 5 nmol per mouse for two weeks. Finally, all the nude mice were killed and the size of the tumours was measured. MiR‐215 agomir and miRNA negative control were purchased from RIBOBIO.

### 2.16 Statistical analysis

The correlations between the module and the tumour phenotype were analysed by Pearson correlation. The log‐rank test was used to compare the survival curves. All experiment data were expressed as the means ± SD. Two‐tailed Student's *t* test was used, and *P* <.05 was considered statistically significant. Statistical analyses were performed on R software (version 3.5.0) and GraphPad Prism software version 7.0 (GraphPad Software).

## 3 RESULTS

### 3.1 Hub‐genes screening analysis

**FIGURE 1:**
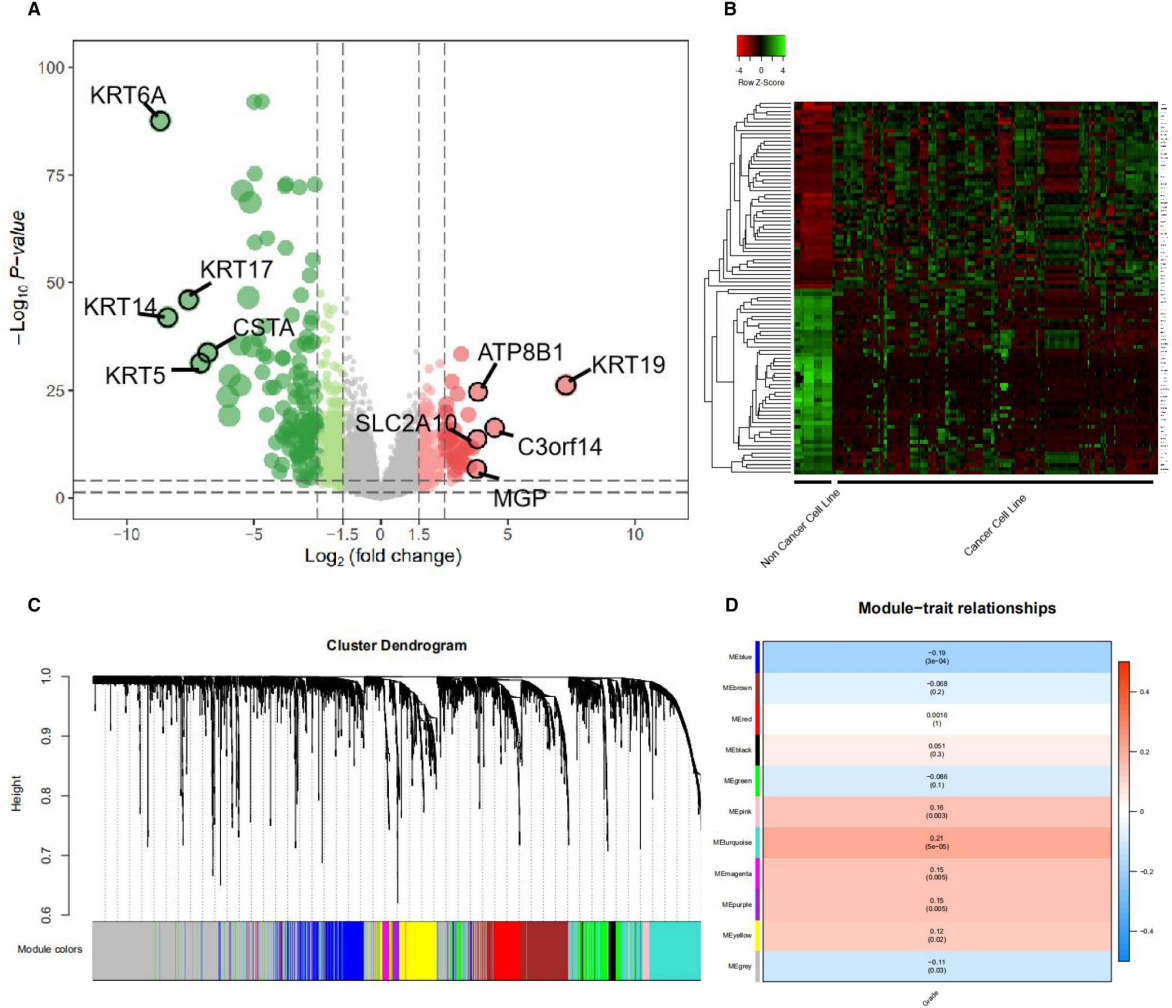
(A) Volcano plot of differentially expressed genes (DEGs) between human breast cancer cell lines and normal breast cells. Red represents genes that are highly expressed in tumour cell lines compared to normal human breast cells, and green represents genes that are lower expressed compared to normal breast cells. (B) Compared with normal human breast cell lines, heat map analysis of the top 50 differentially expressed genes with high and low expression. (C,D) WGCNA analysis of microarray data from 361 clinical BC tissues from the MERAV database. (C) Dendrogram of genes clustered based on a dissimilarity measure (1‐TOM). The colour band shows the results from the automatic single block analysis. (D) Heat map of the correlation between module eigengenes and clinical traits of BC

We used the ‘limma’ package in the R software to analyse the difference in gene expression between BC cell lines and normal breast cells. A volcano map was drawn to show the differences between the two groups (Figure 1A), and an expression heat map was drawn for the 100 genes with the most significant differences (Figure 1B).

For WGCNA analysis, we performed cluster tree analysis on all samples. When the soft threshold *β* = 7 (R^2^ = 0.85), the adjacency coefficient was the lowest, which was the optimal soft threshold for constructing a scale‐free network. Finally, 11 modules were clustered. Each module was distinguished by a different colour, the width of the band matched the number of genes, and the grey band represented genes that were not classified into meaningful modules (Figure 1C). Subsequently, we used WGCNA to explore the correlation of each module with the pathological grading of BC. We found that the turquoise module has the highest correlation with the pathological grade of BC (*r*_p_ = 0.21, *P* = 5×10^−5^, Figure 1D).

### 3.2 Hub‐gene function enrichment and PPI network construction

**FIGURE 2:**
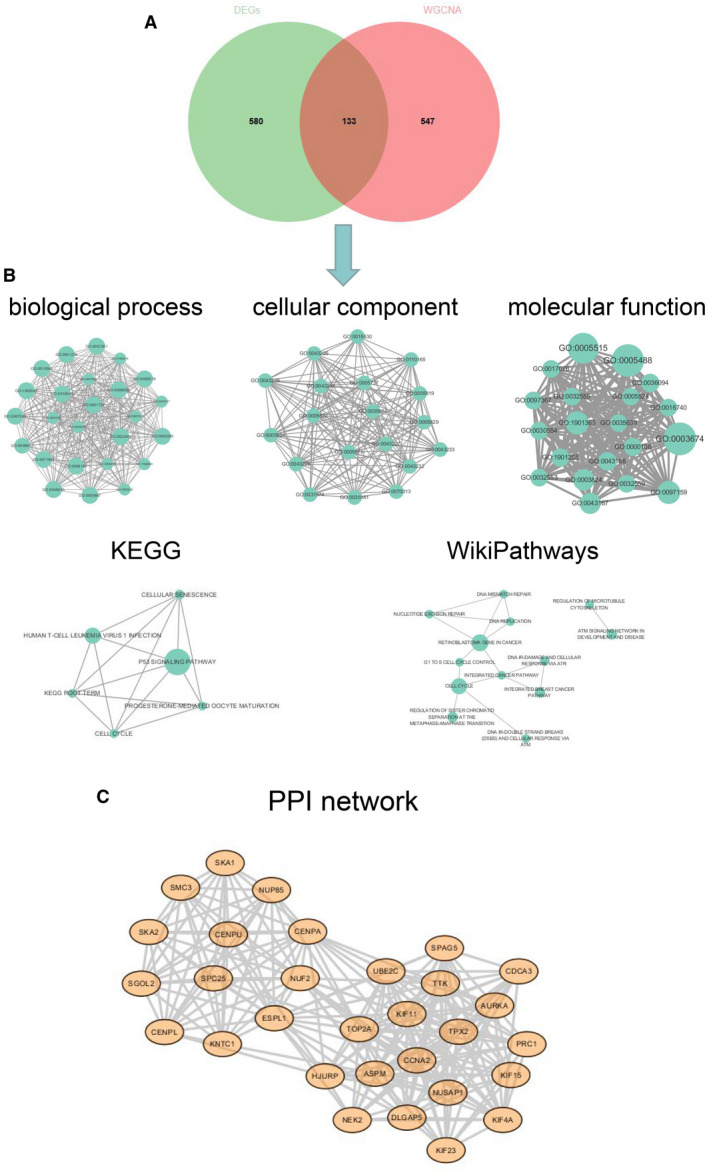
(A) The Venn diagram shows the intersection between the differentially highly expressed genes and the genes of the turquoise module in the WGCNA analysis. For the former, we select differentially highly expressed genes with logFC > 1 and *P* value < .001. For the latter, we select the turquoise module that has the highest correlation with pathological grading. The 133 genes in the intersection are used as data for later analysis. (B) The genes in the intersection were enriched in five aspects: ‘biological process’, ‘cellular component’, ‘molecular function’, ‘KEGG’ and ‘WikiPathways’, and displayed on the network diagram. Among them, it can be clearly seen in the results of KEGG that the p53 signalling pathway was a clear advantage enrichment result. (C) PPI network analysis and extraction of intersection genes. The results show that the closely related networks are mainly divided into two clusters

In order to narrow the screening range, we selected the intersection of differentially highly expressed genes in BC cell lines and the genes in the turquoise module in WGCNA analysis for subsequent analysis. The results showed that there were 133 intersection genes (Figure 2A). The genes in the intersection were enriched in five aspects: ‘biological process’, ‘cellular component’, ‘molecular function’, ‘KEGG’ and ‘WikiPathways’, and displayed on the network diagram. Among them, it can be clearly seen in the results of KEGG that the p53 signalling pathway was a clear advantage enrichment result (Figure 2B). Based on the protein associations obtained from the String database and the utilization of MCODE algorithm, the closely related networks were mainly divided into two clusters (Figure 2C).

### 3.3 Hub‐miRNA mining and integrated database verification

**FIGURE 3:**
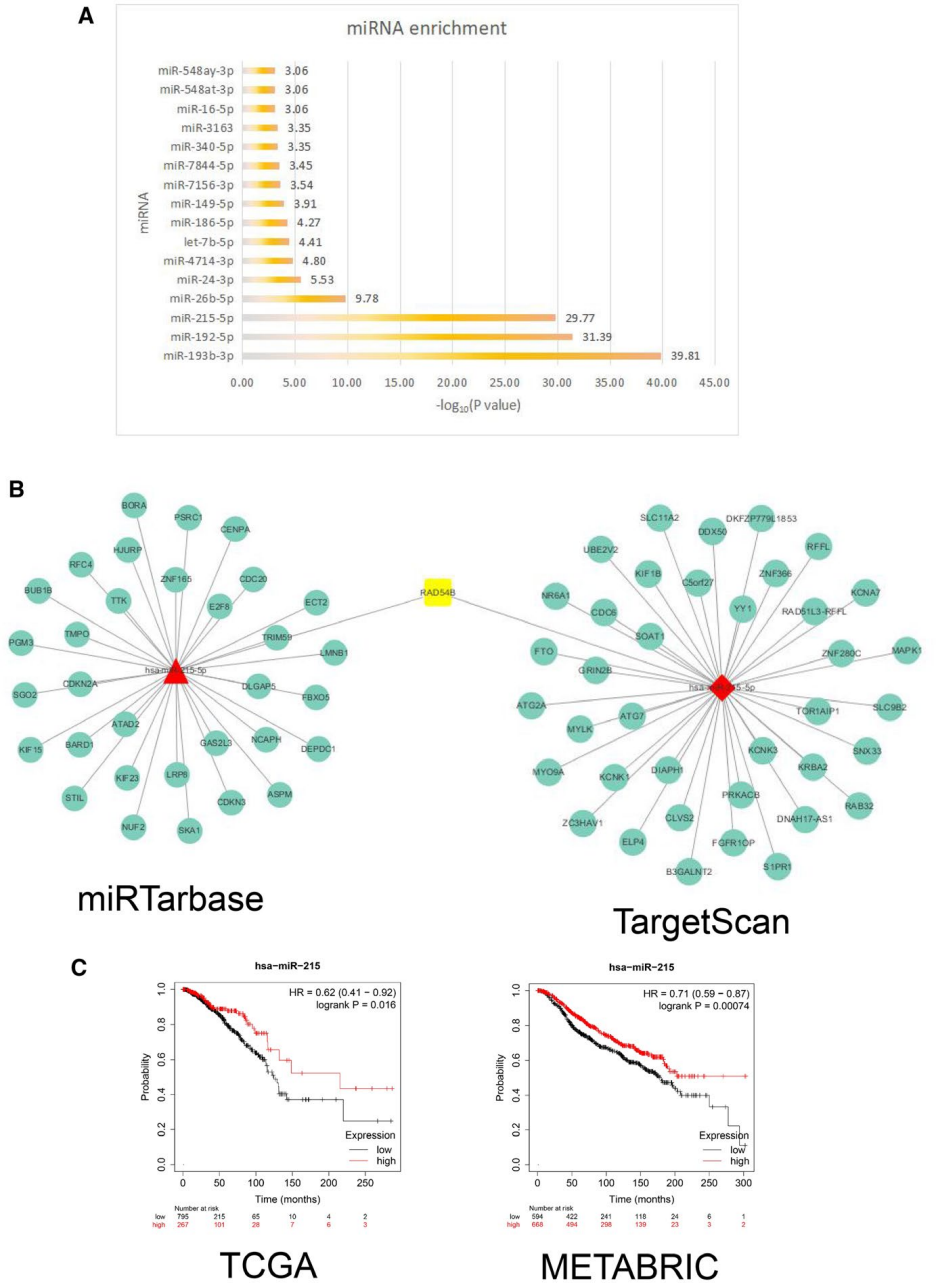
(A) Histogram of miRNA enrichment analysis. The abscissa indicates −log_10_ (*P* value). (B) Prediction of miR‐215‐5p target gene network. The target genes of miR‐215‐5p were predicted in miRTarBase and TargetScan databases respectively. The RAD54B gene not only appeared in the intersection gene in Figure 2, but also appeared in the two prediction results. (C) Kaplan–Meier analysis of overall survival. miR‐215 was used for survival analysis of breast cancer samples in the TCGA database and METABRIC database. The results showed that patients in the group with high miR‐215 expression had a longer survival time

After the intersection genes were targeted for miRNA enrichment, we found that there were three highly enriched miRNAs (miR‐193b‐3p, miR‐192‐5p and miR‐215‐5p, Figure 3A). Subsequently, we combined the two databases (TarBase and TargetScan) to determine the target genes of hub‐miRNAs. As shown in Figure 3B, we found that miR‐215‐5p in the two databases co‐targeted RAD54B.

The expression of miR‐215 in cancer tissues was significantly lower than that in normal tissues (Figure [Link], [Link], [Link]). Furthermore, we found that regardless of the pathological stage, the expression of miR‐215 was significantly lower than that of normal tissues (Figure [Link], [Link], [Link]).

In order to verify the significance of miR‐215 in BC, we selected two BC data sets with clinical information, TCGA and METABRIC, for survival analysis. As shown in Figure 3C, compared to the miR‐215 low expression group, the overall survival time of the miR‐215 high expression group was longer and statistically different. It showed that miR‐215 might act as a protective factor in BC cells.

### 3.4 In vitro experimental verification of miR‐215

**FIGURE 4:**
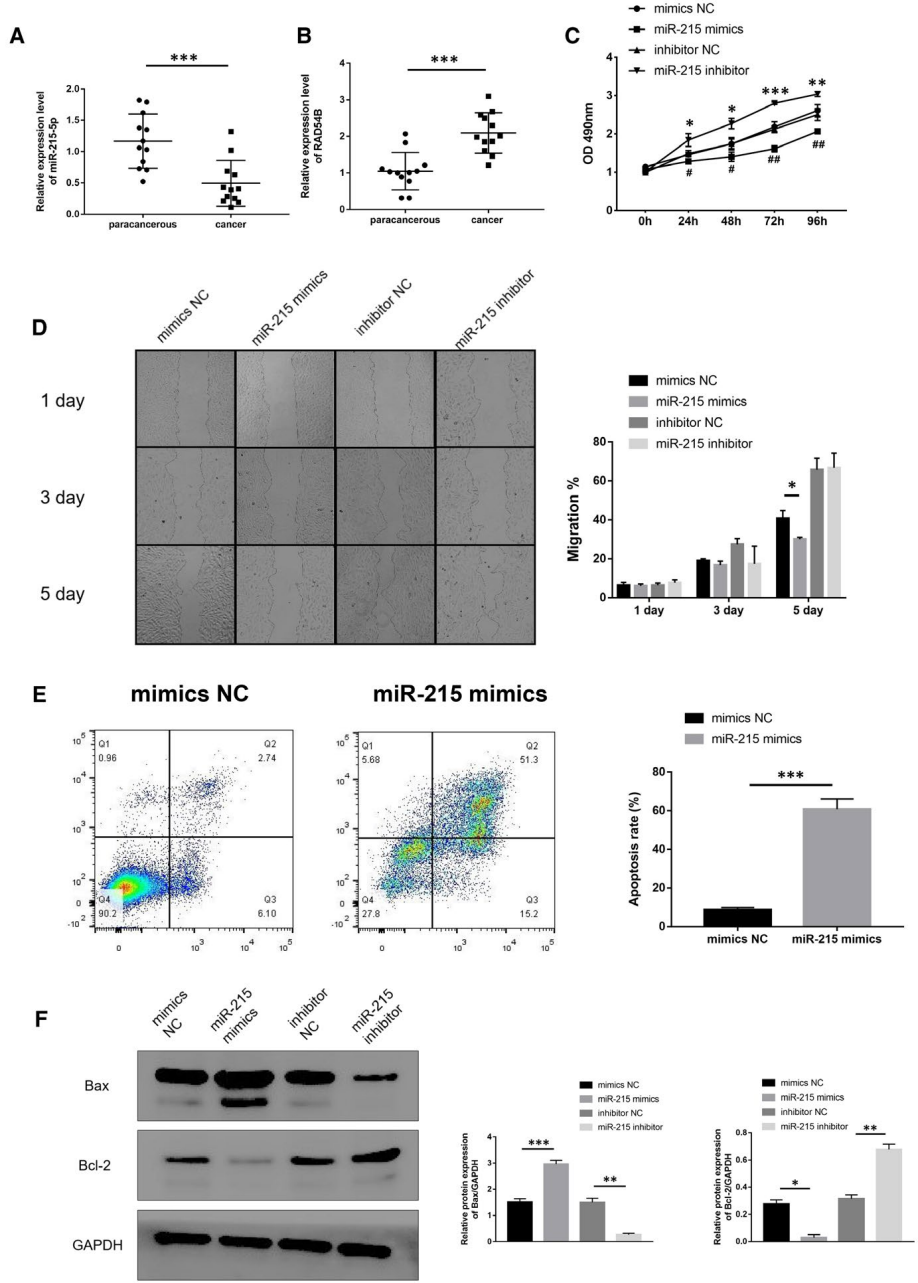
(A) miR‐215‐5p levels in cancer tissues and corresponding adjacent tissues in clinical BC patients were determined by qRT‐PCR. U6 was served as an internal control. (B) RAD54B mRNA levels in cancer tissues and corresponding adjacent tissues in clinical BC patients were determined by qRT‐PCR. GAPDH was served as an internal control. (C) Cell viability of MCF‐7 cancer cell line was monitored by the MTT assay. (D) The migration abilities of mimics NC, miR‐215 mimics, inhibitor NC and miR‐215 inhibitor treated cells were detected by scratch healing experiments. (E) Apoptosis was determined by flow cytometry. MCF‐7 cells were treated by mimics NC and miR‐215 mimics separately. (F) Protein levels of apoptosis‐related genes Bax and Bcl‐2 were determined by western blotting. GAPDH served as loading control. Data were representative images or were expressed as the mean ± SD of n = 3 experiments. *P < .05, **P < .01 and ***P < .001

Quantitative PCR detection was performed on total RNA extracted from specimens of 12 clinical BC patients. The results showed that compared with normal adjacent tissues, the expression of miR‐215‐5p in tumour tissues was lower and statistically different (Figure 4A). Correspondingly, the mRNA expression of RAD54B had an opposite trend (Figure 4B).

After up‐regulating the expression of miR‐215 in the BC cell line MCF‐7, the cell proliferation rate was significantly reduced compared to the control group. After inhibiting miR‐215, the rate of cell proliferation increased significantly (Figure 4C). This indicated that miR‐215 can inhibit cell proliferation.

Furthermore, we used the scratch healing test to verify the effect of miR‐215 on the migration phenotype of BC cell lines. Similarly, we found that after overexpression of miR‐215, compared with the control group, the migration of MCF‐7 was significantly inhibited on the fifth day (Figure 4D).

Subsequently, we collected the cells of the miR‐215 mimics group and the mimics NC group, and used flow cytometry to detect the apoptosis of the cells. We found that the apoptotic rate of miR‐215 overexpression group increased significantly on the fifth day (Figure 4E). This suggested that miR‐215 inhibits BC cell proliferation and migration may be related to the increase in apoptosis rate.

To further illustrate that miR‐215 can affect the apoptosis level of BC cells. We applied Western blotting to detect the expression of apoptosis‐related proteins BCL2 Associated X (Bax) and BCL2 Apoptosis Regulator (Bcl‐2) in MCF‐7 cells. Compared with the mimics NC group, after standardization with the internal reference protein GAPDH, the protein expression level of Bax in the miR‐215 mimics group was significantly increased, and the protein expression level of Bcl‐2 was significantly reduced. On the other hand, compared with the inhibitor NC group, after standardization with the internal reference protein GAPDH, the protein expression level of Bax in the miR‐215 inhibitor group was significantly reduced, and the protein expression level of Bcl‐2 was significantly increased (Figure 4F). This result further showed that miR‐215 could up‐regulate the apoptosis level of BC cells.

### 3.5 miR‐215 targets RAD54B to promote BC cell apoptosis

**FIGURE 5:**
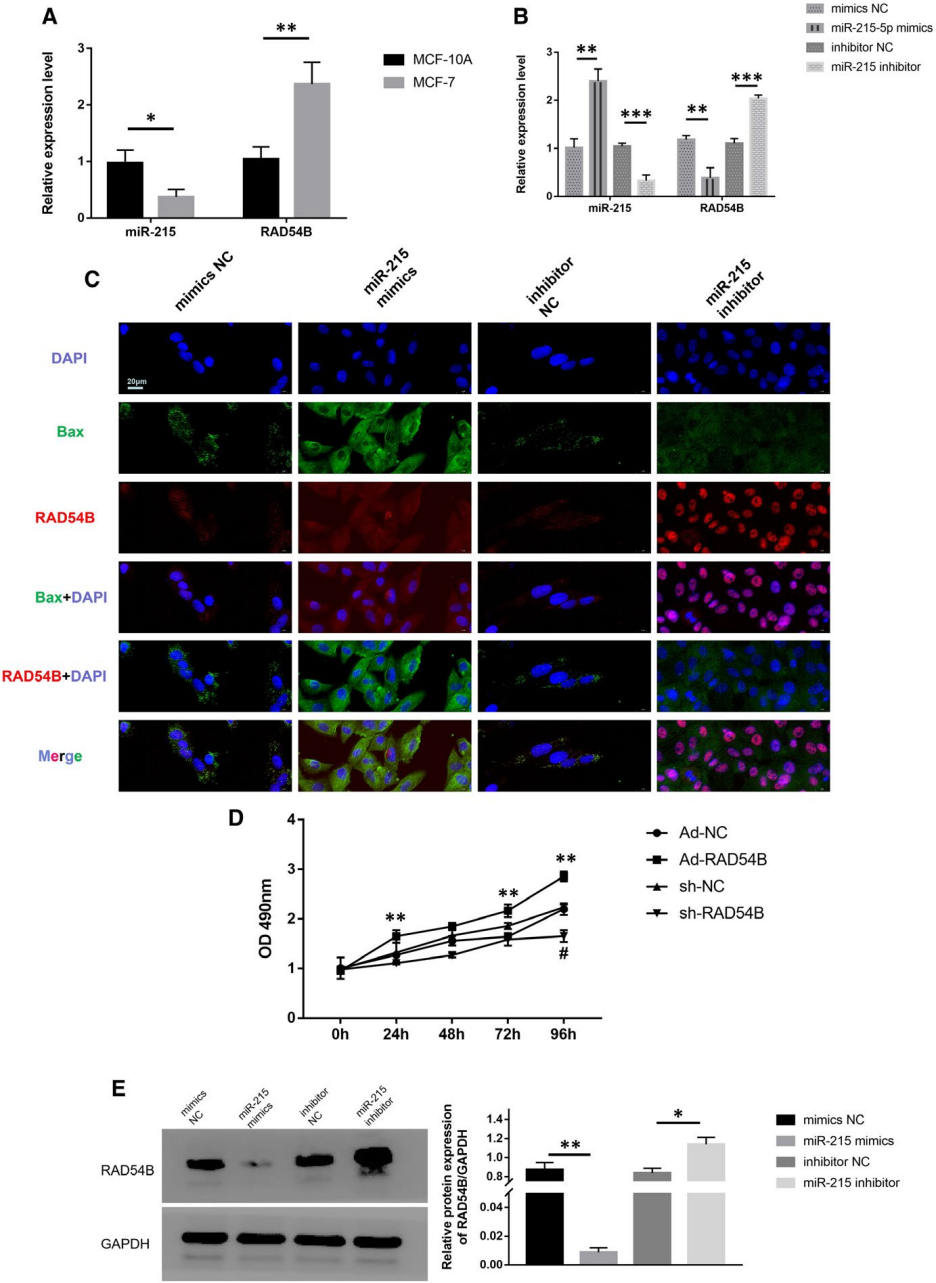
(A) miR‐215 and RAD54B mRNA levels in MCF‐10A and MCF‐7 were determined by qRT‐PCR. U6 or GAPDH served as an internal control. (B) miR‐215 and RAD54B mRNA levels in MCF‐7 treated by mimics NC, miR‐215 mimics, inhibitor NC and miR‐215 inhibitor were determined by qRT‐PCR. U6 or GAPDH served as an internal control. (C) Immunofluorescence analysis of RAD54B and Bax in MCF‐7 cells treated by mimics NC, miR‐215 mimics, inhibitor NC and miR‐215 inhibitor. The nuclei were stained with DAPI. Scale bar = 20 μm. (D) Cell viability of MCF‐7 treated by Ad‐NC, Ad‐RAD54B, sh‐NC and sh‐RAD54B was monitored by the MTT assay. (E) Protein levels of RAD54B in MCF‐7 cells treated by mimics NC, miR‐215 mimics, inhibitor NC and miR‐215 inhibitor were determined by western blotting. GAPDH served as loading control. Data were representative images or were expressed as the mean ± SD of n = 3 experiments. *P < .05, **P < .01 and ***P < .001

In order to explore the correlation between miR‐215 and RAD54B, we first compared the difference between miR‐215 and RAD54B mRNA in BC cell line MCF‐7 and normal breast cell MCF‐10A. Quantitative PCR results showed that compared with MCF‐10A, miR‐215 in MCF‐7 showed a low expression trend, while RAD54B mRNA showed a high expression trend (Figure 5A). Subsequently, we overexpressed miR‐215 in MCF‐7 and found that the expression of RAD54B mRNA was suppressed. Conversely, when miR‐215 was inhibited, the expression level of RAD54B increased significantly (Figure 5B). This indicated that the levels of miR‐215 and RAD54B mRNA were negatively correlated.

We performed IF staining on MCF‐7 cells. The results showed that the Bax (green fluorescence) signal intensity of the miR‐215 mimics group was more dominant than the mimics NC group. The RAD54B (red fluorescence) is relatively dim. On the other hand, compared to the inhibitor NC group, the red fluorescence signal of the miR‐215 inhibitor group was more significant, while the green fluorescence signal was significantly reduced (Figure 5C). Cell proliferation experiments showed that compared with the control group, the cell proliferation rate of MCF‐7 cells in the RAD54B overexpression group was significantly increased at 24, 72, and 96 hours. The cell proliferation rate of the RAD54B down‐regulated group was inhibited at 96 hours (Figure 5D). This showed that miR‐215, RAD54B and apoptosis have a certain degree of causality.

When we overexpressed miR‐215 in MCF‐7 cells, we found that the protein level of RAD54B was significantly suppressed. Conversely, when miR‐215 was inhibited, the protein level of RAD54B was significantly restored (Figure 5E). This indicates that miR‐215 may inhibit the protein expression of RAD54B mRNA by inhibiting its translation.

Furthermore, we added the recovery test to further verify the relationship between miR‐215 and RAD54B. After overexpressing miR‐215 (miR‐215 mimics) in BC cells, the MTT method detected that the cell proliferation was significantly inhibited. However, when miR‐215 and RAD54B (miR‐215 mimics + Ad‐RAD54B) were overexpressed at the same time, the cell proliferation phenotype was significantly restored (Figure [Link], [Link], [Link]).

**FIGURE 6:**
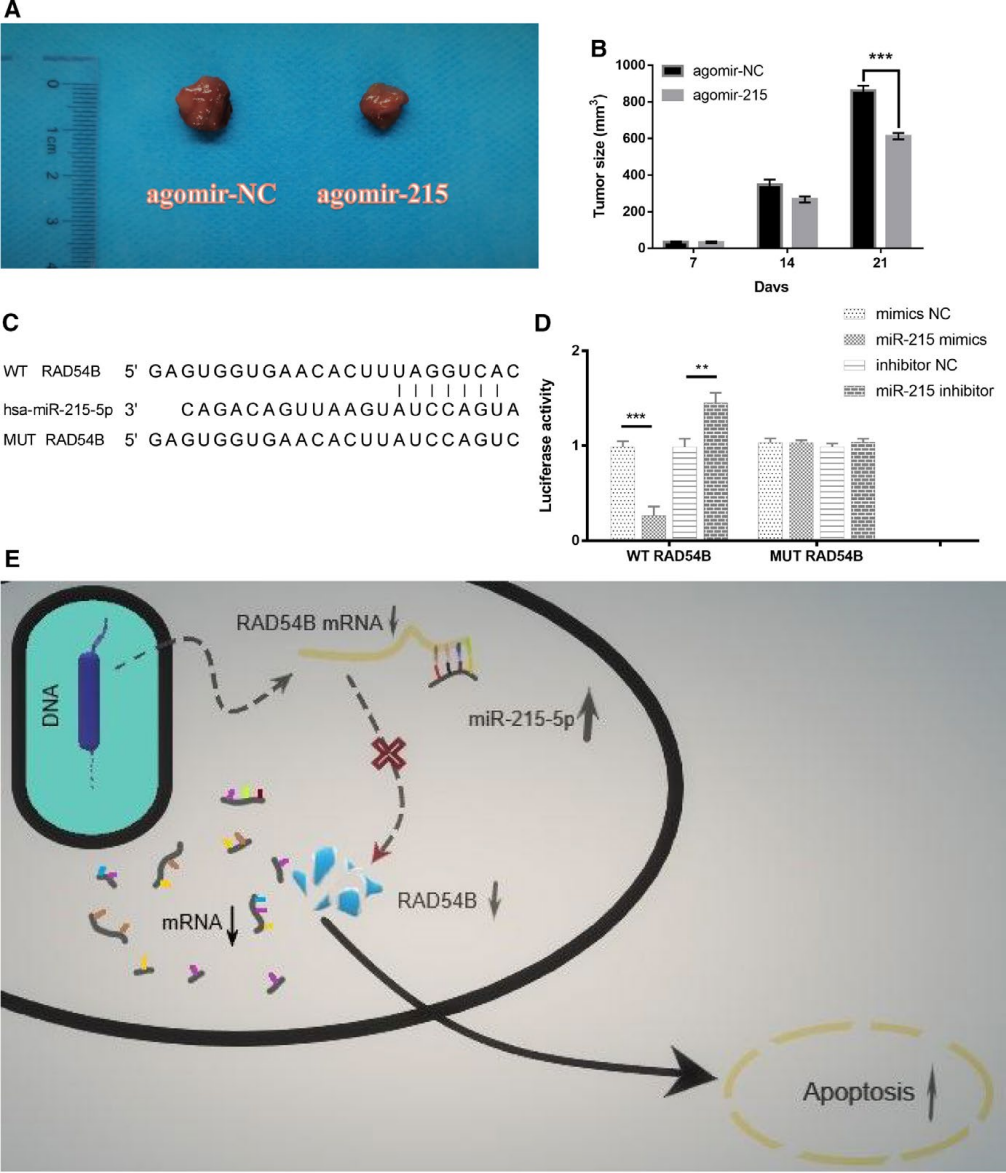
(A) Photographs of tumour xenografts from the agomir‐215 and agomir‐NC groups. (B) Histogram of tumour size in different groups. Data were representative images. n = 3 experiments and ***P < .001. (C) The predicted binding sites between miR‐215‐5p and RAD54B 3′‐UTR. A mutation was generated in 3′‐UTR of RAD54B in the complementary site for miR‐215‐5p binding. (D) Cotransfection of WT/MUT RAD54B 3′‐UTR and mimics NC/miR‐215 mimics/inhibitor NC/miR‐215 inhibitor in HEK293T cells. Firefly luciferase activity was examined by dual luciferase reporter assay. Renilla luciferase activity was used to normalize the activity of firefly luciferase. n = 3 experiments, **P < .01 and ***P < .001. (E) A schematic diagram illustrating that miR‐215‐5p inhibits the translation of RAD54B mRNA and induces apoptosis by binding to the 3′UTR region of RAD54B mRNA

For in vivo studies in nude mice, we found that overexpression of miR‐215 was conducive to limiting tumour growth. Compared with the negative control group, the tumour formation in nude mice overexpressing miR‐215 were significantly reduced. This shows that miR‐215 has an inhibitory effect on BC and at the same time confirms the correctness of our previous results (Figure 6A,B).

Next, we hoped to prove that RAD54B was a direct target of miR‐215. Bioinformatics analysis revealed that the 3′UTR of RAD54B and miR‐215‐5p had completely complementary paired fragments. The results of the dual luciferase reporter gene showed that the luciferase activity of cotransfected miR‐215 mimics and WT RAD54B was significantly inhibited, but there was no significant difference in the luciferase activity of the MUT RAD54B group. In contrast, the cotransfected miR‐215 inhibitor and WT RAD54B luciferase activity were activated, but there was no significant difference in the luciferase activity in the MUT RAD54B group. This showed that miR‐215 could directly target RAD54B mRNA and inhibited its expression level (Figure 6C,D). Figure 6E shows a schematic diagram of miR‐215 targeting RAD54B and inducing apoptosis.

## 4 DISCUSSION

BC is the most common malignant tumour in women. Studies have shown that the abnormal expression of miRNAs is closely related to the occurrence of BC, and play important roles in tumour generation, differentiation, invasion and metastasis.^20^, ^21^ The mature miRNA can degrade the target sequence or inhibit post‐transcriptional translation through complete or incomplete complementary pairing with the target gene sequence, and finally induce gene silencing. Most miRNAs are highly conservative, sequential and tissue specific, and are important parts of normal cell function, but abnormal expression can also promote human diseases. miRNAs play important roles in many malignant tumours. Recent studies have found that miRNAs related to breast cancer, including let‐7, miR‐21, miR‐10b, miR‐17‐5p, miR‐155, miR‐145 and miR‐520b, act as oncogenes or tumour suppressor genes, and promote or inhibit the occurrence, development and metastasis of tumours.^22^, ^23^ The regulatory network of miRNAs and target genes is extremely precise. Once the network balance is broken, there will be disordered genes expression, followed by the occurrence of tumours or other diseases. In order to better understand the mechanism of miRNAs, it is necessary to know which miRNA acts on which gene. In this study, we tried to identify the key miRNAs and their target genes in BC through bioinformatics analysis, and explore their specific molecular mechanisms through experiments. We found that miR‐215 may be a potential key molecule with regulatory effects in BC cells. MiR‐215 inhibits the protein expression of RAD54B by targeting the 3′UTR of RAD54B mRNA, thereby inducing BC cells apoptosis. This discovery may reveal new ways of regulation in BC cells and improve the network mechanism of miRNA and target gene regulation.

Studies have found that miR‐215 was associated with occurrence of metastases and survival in BC.^24^, ^25^ High expression of miR‐215 reduced proliferation and migration of cells.^26^, ^27^ It has been proved that miR‐215‐5p was lower expressed in BC compared to normal controls, even demonstrating anti‐tumorigenic effects in vivo.^28^ The above researches are consistent with the conclusion of this study, which further illustrates the correctness of our conclusion.

RAD54 Homolog B (RAD54B) is a cofactor of RAD51 and plays a role in homologous recombination repair of DNA breaks, which may affect cell survival and genome stability. Homozygous mutations in highly conserved positions of RAD54B have been found in human primary lymphoma and colon cancer.^29^ Using a RAD54B‐deficient human colon cancer cell line, Miyagawa et al found that RAD54B plays a key role in the sensitivity of DNA damaging agents or the integration of sister chromatid exchange.^30^ In addition, inhibiting RAD54B can inhibit the proliferation of liver cancer cells and promote cell apoptosis.^31^ Our study found that RAD54B can be used as a key factor in promoting BC proliferation and worsening the prognosis. This is consistent with previous researches conclusions. Furthermore, this lays the foundation for further experimental research.

The regulation mechanism of internal molecules in tumour cells is complicated. The screening of key molecules and the exploration of mechanisms are conducive to accurately reveal the characteristics of tumours and provide evidence‐based clues for the selection of treatment options. Bioinformatics data analysis combined with molecular biology experiments has increasingly become the main method to discover the key factors of diseases. In this study, we tried to explore the complex phenotypic characteristics of the disease by looking for individual molecules. We elucidate the critical role of miR‐215/RAD54B axis in BC, which provides reference value for perfecting the regulatory role of miRNAs in tumours.

## 5 CONCLUSION

In summary, our research shows that miR‐215 can induce the activation of BC cell apoptosis pathway and inhibit the proliferation and migration of BC cells by inhibiting the protein expression of RAD54B, thereby improving the prognosis of BC patients. This finding clarifies the significance of the miR‐215/RAD54B/apoptotic pathway axis and implies the potential application of miR‐215 in the treatment of BC.

## CONFLICT OF INTEREST

All authors declare that they have no conflict of interest.

## AUTHOR CONTRIBUTION

**Mingyuan Wang:** Conceptualization (lead). **Jingnan Liao:** Data curation (supporting). **Chang Tan:** Methodology (supporting). **Hong Zhou:** Methodology (supporting). **Jinjin Wang:** Methodology (supporting). **Kangkai Wang:** Conceptualization (supporting). **Yiming Li:** Writing‐review & editing (supporting). **Wei Wu:** Conceptualization (equal).

## ETHICS APPROVAL AND CONSENT TO PARTICIPATE

The research protocol was approved by the Ethics Committee of Zhuzhou Central Hospital, Central South University (Zhuzhou, China). Informed consent was obtained from all subjects.

## Supporting information

Fig S1

Fig S2

Fig S3

## Data Availability

The data that support the findings of this study are openly available in the MERAV (MERAV, http://merav.wi.mit.edu) database.

## References

[1] Adhikary S , Chakravarti D , Terranova C , et al. Atypical plant homeodomain of UBR7 functions as an H2BK120Ub ligase and breast tumor suppressor. Nat Commun. 2019;10:1398. 30923315 10.1038/s41467-019-08986-5 PMC6438984

[2] Chen X , Ariss MM , Ramakrishnan G , et al. Cell‐autonomous versus systemic Akt isoform deletions uncovered new roles for Akt1 and Akt2 in breast cancer. Mol Cell. 2020;80:87‐101. 32931746 10.1016/j.molcel.2020.08.017 PMC8291754

[3] Aganezov S , Goodwin S , Sherman RM , et al. Comprehensive analysis of structural variants in breast cancer genomes using single‐molecule sequencing. Genome Res. 2020;30:1258‐1273. 32887686 10.1101/gr.260497.119 PMC7545150

[4] Beesley J , Sivakumaran H , Moradi MM , et al. eQTL colocalization analyses identify NTN4 as a candidate breast cancer risk gene. Am J Hum Genet. 2020;107:778‐787. 32871102 10.1016/j.ajhg.2020.08.006 PMC7536644

[5] Nana‐Sinkam SP , Hunter MG , Nuovo GJ , et al. Integrating the MicroRNome into the study of lung disease. Am J Respir Crit Care Med. 2009;179:4‐10. 18787215 10.1164/rccm.200807-1042PP PMC2615660

[6] Miele E , Buttarelli FR , Arcella A , et al. High‐throughput microRNA profiling of pediatric high‐grade gliomas. Neuro Oncol. 2014;16:228‐240. 24305714 10.1093/neuonc/not215 PMC3895388

[7] Capizzi M , Strappazzon F , Cianfanelli V , et al. MIR7‐3HG, a MYC‐dependent modulator of cell proliferation, inhibits autophagy by a regulatory loop involving AMBRA1. Autophagy. 2017;13:554‐566. 28059583 10.1080/15548627.2016.1269989 PMC5361610

[8] Leng X , Huang G , Ding J , et al. Circ_0000043 promotes breast cancer cell proliferation, migration, invasion and epithelial‐mesenchymal transition via the miR‐136/ Smad3 axis. Biochem Cell Biol. 2020. Online ahead of print. 10.1139/bcb-2020-0219 33043682

[9] Lim S , Kim Y , Lee SB , et al. Inhibition of Chk1 by miR‐320c increases oxaliplatin responsiveness in triple‐negative breast cancer. Oncogenesis. 2020;9:91. 33041328 10.1038/s41389-020-00275-x PMC7548284

[10] Yang W , Feng W , Wu F , et al. MiR‐135‐5p inhibits TGF‐beta‐induced epithelial‐mesenchymal transition and metastasis by targeting SMAD3 in breast cancer. J Cancer. 2020;11:6402‐6412. 33033523 10.7150/jca.47083 PMC7532519

[11] Wang M , Wang J , Liu J , et al. Systematic prediction of key genes for ovarian cancer by co‐expression network analysis. J Cell Mol Med. 2020;24:6298‐6307. 32319226 10.1111/jcmm.15271 PMC7294139

[12] Wang M , Li L , Liu J , et al. A gene interaction networkbased method to measure the common and heterogeneous mechanisms of gynecological cancer. Mol Med Rep. 2018;18:230‐242. 29749503 10.3892/mmr.2018.8961 PMC6059674

[13] Shaul YD , Yuan B , Thiru P , et al. MERAV: a tool for comparing gene expression across human tissues and cell types. Nucleic Acids Res. 2016;44:D560‐D566. 26626150 10.1093/nar/gkv1337 PMC4702927

[14] Wang M , Liao J , Wang J , et al. TAF1A and ZBTB41 serve as novel key genes in cervical cancer identified by integrated approaches. Cancer Gene Ther. 2020. Online ahead of print. 10.1038/s41417-020-00278-1 PMC8636252 33311601

[15] Raudvere U , Kolberg L , Kuzmin I , et al. g:Profiler: a web server for functional enrichment analysis and conversions of gene lists (2019 update). Nucleic Acids Res. 2019;47:W191‐W198. 31066453 10.1093/nar/gkz369 PMC6602461

[16] Szklarczyk D , Gable AL , Lyon D , et al. STRING v11: protein‐protein association networks with increased coverage, supporting functional discovery in genome‐wide experimental datasets. Nucleic Acids Res. 2019;47:D607‐D613. 30476243 10.1093/nar/gky1131 PMC6323986

[17] Nagy A , Lanczky A , Menyhart O , et al. Validation of miRNA prognostic power in hepatocellular carcinoma using expression data of independent datasets. Sci Rep. 2018;8:9227. 29907753 10.1038/s41598-018-27521-y PMC6003936

[18] Chandrashekar DS , Bashel B , Balasubramanya SAH , et al. UALCAN: a portal for facilitating tumor subgroup gene expression and survival analyses. Neoplasia. 2017;19(8):649‐658. 28732212 10.1016/j.neo.2017.05.002 PMC5516091

[19] Jiang B , Tang Y , Wang H , et al. Down‐regulation of long non‐coding RNA HOTAIR promotes angiogenesis via regulating miR‐126/SCEL pathways in burn wound healing. Cell Death Dis. 2020;11:61. 31974341 10.1038/s41419-020-2247-0 PMC6978466

[20] Andorfer CA , Necela BM , Thompson EA , et al. MicroRNA signatures: clinical biomarkers for the diagnosis and treatment of breast cancer. Trends Mol Med. 2011;17:313‐319. 21376668 10.1016/j.molmed.2011.01.006

[21] Al‐Othman N , Ahram M , Alqaraleh M . Role of androgen and microRNA in triple‐negative breast cancer. Breast Dis. 2020;39:15‐27. 31839601 10.3233/BD-190416

[22] Tang J , Ahmad A , Sarkar FH . MicroRNAs in breast cancer therapy. Curr Pharm Des. 2014;20:5268‐5274. 24479805 10.2174/1381612820666140128205239

[23] Kanchan RK , Siddiqui JA , Mahapatra S , et al. microRNAs orchestrate pathophysiology of breast cancer brain metastasis: advances in therapy. Mol Cancer. 2020;19:29. 32059676 10.1186/s12943-020-1140-x PMC7023699

[24] Madhavan D , Peng C , Wallwiener M , et al. Circulating miRNAs with prognostic value in metastatic breast cancer and for early detection of metastasis. Carcinogenesis. 2016;37:461‐470. 26785733 10.1093/carcin/bgw008

[25] Zhou SW , Su BB , Zhou Y , et al. Aberrant miR‐215 expression is associated with clinical outcome in breast cancer patients. Med Oncol. 2014;31:259. 25270284 10.1007/s12032-014-0259-2

[26] Leblanc N , Harquail J , Crapoulet N , et al. Pax‐5 inhibits breast cancer proliferation through MiR‐215 up‐regulation. Anticancer Res. 2018;38:5013‐5026. 30194145 10.21873/anticanres.12820

[27] Yao J , Zhang P , Li J , et al. MicroRNA‐215 acts as a tumor suppressor in breast cancer by targeting AKT serine/threonine kinase 1. Oncol Lett. 2017;14:1097‐1104. 28693279 10.3892/ol.2017.6200 PMC5494665

[28] Gao JB , Zhu MN , Zhu XL . miRNA‐215‐5p suppresses the aggressiveness of breast cancer cells by targeting Sox9. Febs Open Bio. 2019;9:1957‐1967. 10.1002/2211-5463.12733 PMC6823282 31538724

[29] Hiramoto T , Nakanishi T , Sumiyoshi T , et al. Mutations of a novel human RAD54 homologue, RAD54B, in primary cancer. Oncogene. 1999;18:3422‐3426. 10362364 10.1038/sj.onc.1202691

[30] Miyagawa K , Tsuruga T , Kinomura A , et al. A role for RAD54B in homologous recombination in human cells. Embo J. 2002;21:175‐180. 11782437 10.1093/emboj/21.1.175 PMC125815

[31] Wang R , Li Y , Chen Y , et al. Inhibition of RAD54B suppresses proliferation and promotes apoptosis in hepatoma cells. Oncol Rep. 2018;40:1233‐1242. 29956808 10.3892/or.2018.6522 PMC6072389

