# Supplementary figures and images for: Integrated study of miR‐215 promoting breast cancer cell apoptosis by targeting RAD54B
Source: J Cell Mol Med. 2021 Feb 26;25(7):3327–38. doi: 10.1111/jcmm.16402 (PMC8034472; doi:10.1111/jcmm.16402)

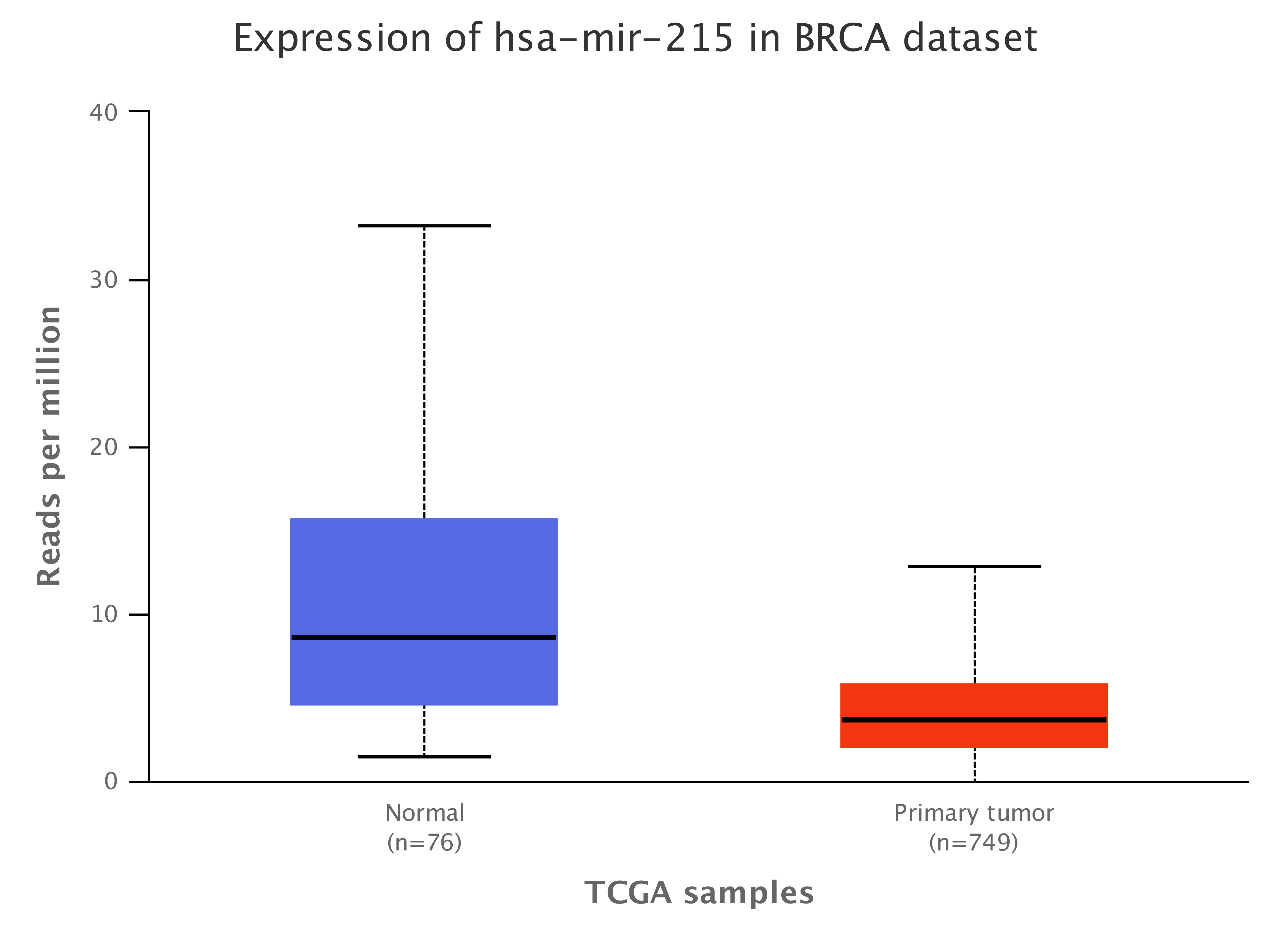

Supplement: Supplementary file 1 — Fig S1 [file JCMM-25-3327-s001.jpg]

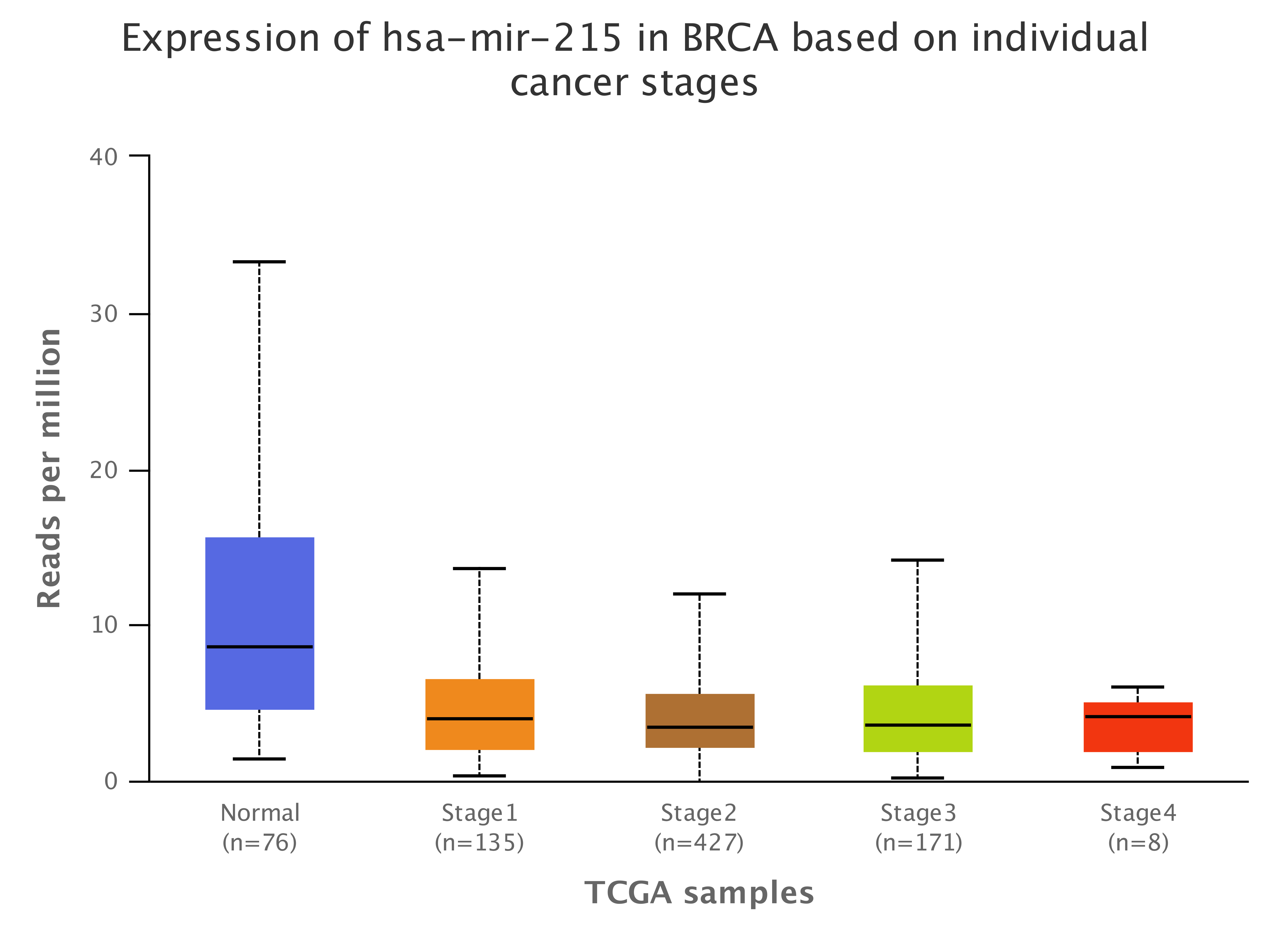

Supplement: Supplementary file 2 — Fig S2 [file JCMM-25-3327-s002.jpg]

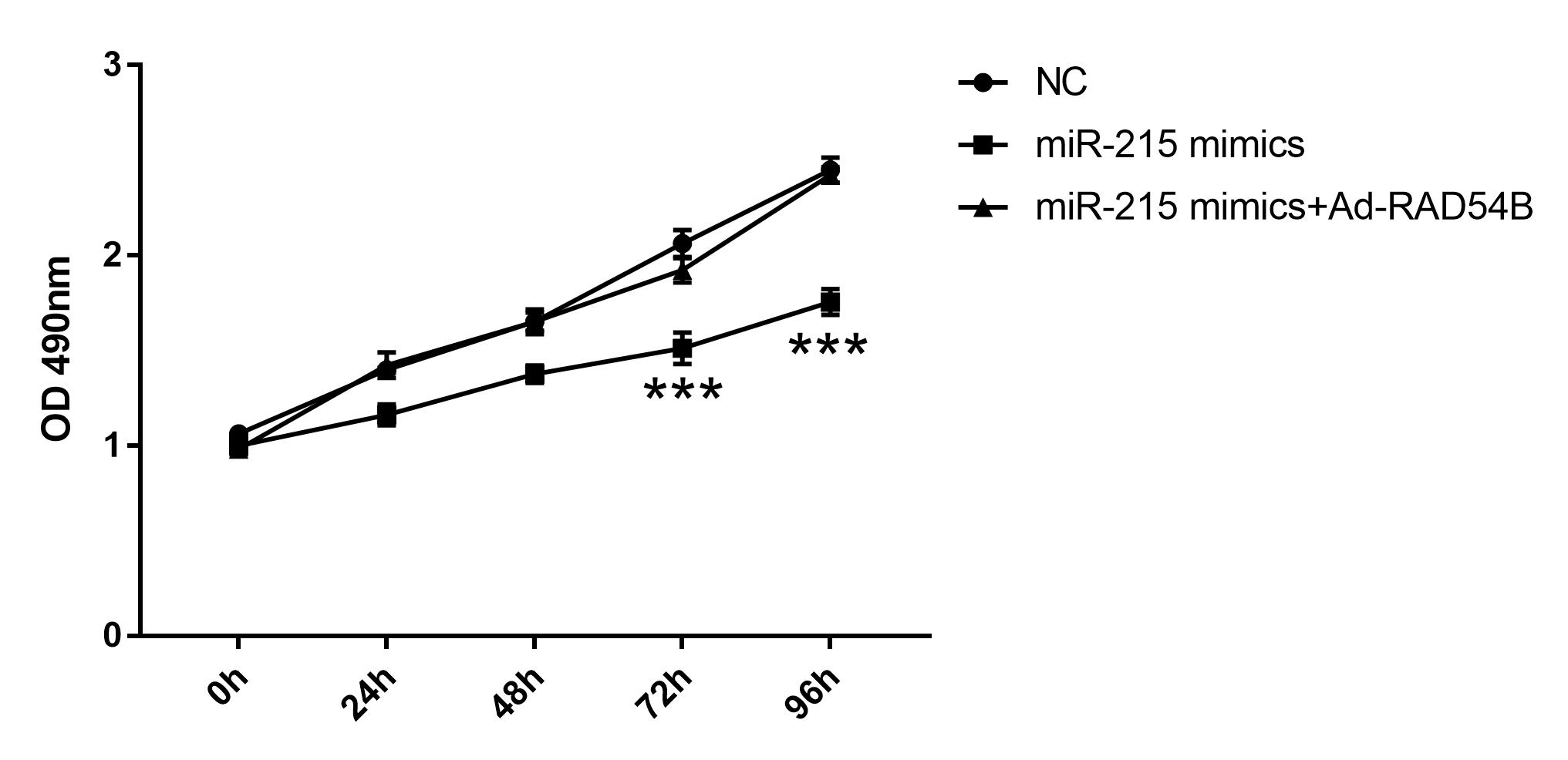

Supplement: Supplementary file 3 — Fig S3 [file JCMM-25-3327-s003.jpg]
